# Remote Ischemic Conditioning in Acute Ischemic Stroke – A Clinical Trial Design

**DOI:** 10.25122/jml-2020-0049

**Published:** 2020

**Authors:** Alina Poalelungi, Elena Turiac, Delia Tulbă, Diana Stoian, Bogdan Ovidiu Popescu

**Affiliations:** 1. Neurology Department, Emergency Clinical Hospital Bucharest, Bucharest, Romania; 2. Radiology Department, Emergency Clinical Hospital Bucharest, Bucharest, Romania; 3. Neurology Department, Colentina Clinical Hospital, Bucharest, Romania; 4. Carol Davila University of Medicine and Pharmacy, Bucharest, Romania; 5. “Victor Babes” National Institute of Pathology, Bucharest, Romania

**Keywords:** Remote ischemic conditioning, acute ischemic stroke, neuroprotection, infarct size

## Abstract

Remote ischemic conditioning represents an intervention based on blood flow reduction applied at a distance from the lesion. The mechanism is supposed to elicit neurovascular protection, anti-inflammatory action, reduced excitotoxicity and metabolic protection. This study aims to explore the efficiency and safety of remote ischemic conditioning during the first five days following in patients who are ineligible for reperfusion treatment (intravenous thrombolysis or/and mechanical thrombectomy). We hypothesized that this intervention would reduce the infarct size (neuroprotection in the reperfusion window) and improve functional recovery. We aim to conduct a double-blind controlled trial, multicenter in two hospitals in Romania. Two hundred patients with acute ischemic stroke randomly divided into an experimental group and a control group will be included. The subjects in the experimental group will be subjected to remote ischemic conditioning twice daily with a maximum of 180 mmHg for 5 days, and a guideline- based treatment as well. The subjects in the control group will receive cuff inflation to 30 mmHg, which will induce sham preconditioning. The primary outcome measure will be radiological - the difference between baseline brain infarct volume and the volume at 180 days in the experimental group versus the control group. The second outcome considers clinical scores such as NIHSS, mRS, IADL, ADL, MOCA, PHQ-9 at baseline, 90 and 180 days; tolerance and side effects of remote ischemic conditioning; the reccurence of stroke or other vascular events at 180 days; incidence of stroke-associated comorbidities and the proportion of death of any cause within 180 days.

## Introduction

Ischemic stroke is the leading cause of death and disability worldwide, being a socioeconomic problem in the 21st century, despite the treatment advances. According to the World Health Organization, stroke is the second cause of death after neoplasia. The incidence is variable depending on the country, region, and quality of medical assistance. The incidence rate of ischemic stroke among young people has increased in recent years [1].

In Romania, stroke is a public health problem, with more than 60.000 new strokes occurring every year and 200 new strokes daily. Accordingly, every 10 minutes, there is a new stroke case. One-third of these patients remain with disabilities.

Most strokes could be prevented by an efficient system of prevention, which would include strategies to educate the community about modifiable risk factors and the warning signs of a stroke.

The INTERSTROKE study – a standardized case-control study – found that hypertension, physical inactivity, poor diet, diabetes, abdominal obesity, current smoking, alcohol consumption, psychosocial factors, cardiac causes, waist to hip ratio, apolipoprotein ratio, are associated with 90.7% of all strokes [2].

The current recommended treatment for acute ischemic stroke (AIS) includes reperfusion treatment (intravenous thrombolysis, mechanical thrombectomy) and aspirin. In Romania, the reperfusion treatment has not peaked at its full potential yet. Only a small part of the population can receive reperfusion treatment due to the limited window, technical requirements and lack of recognizing stroke signs. A substantial number of stroke patients remain with a physical disability, and the burden of stroke is massive, with a significant negative impact on patients and caregivers as well as the socioeconomic status. At this moment, there is no alternative treatment available.

In the last years, some neuroprotective studies conducted on animals with good results have been published, but similar research failed in the clinical trials.

Remote ischemic conditioning (RIC) is an intervention that reduces the blood flow distally from an ischemic lesion in an attempt to minimize the ischemic injury of the organ (in stroke, in a tissue different than the brain). A mechanical tourniquet placed around a standard arm or leg will perform repetitive inflation and deflation. The mechanisms underlying this effect are neurovascular protection, anti-inflammatory action, reduced excitotoxicity, and metabolic protection [3]. RIC is a potential neuroprotective new treatment that has been demonstrated as being safe when translating from animals to clinical trials. Nevertheless, the few trials assessing its efficiency in reducing the ischemic in the injury of the brain have inconsistent results.

This study aims to explore the efficiency and safety of RIC to the arm during the first five days following AIS without the reperfusion treatment (intravenous thrombolysis, mechanical thrombectomy). We hypothesized here that RIC would reduce the infarct size (neuroprotection in the reperfusion window) and improve functional recovery.

## Material and Methods

### Design

This is a double-blind, randomized controlled multicenter trial that is performed in two hospitals from Romania (Emergency Clinical Hospital and Colentina Hospital, both located in Bucharest). It is conducted according to medical bioethics, respecting the ethical principles for medical research involving human subjects – World Medical Association (Helsinki). The study was approved by an institutional review committee, and the subjects gave full informed consent. All the patients will receive standard treatment for secondary prevention of AIS according to the guidelines of the Romanian Society of Neurology (antiplatelets, blood pressure control and cholesterol reduction therapy).

### Patient population

A total of 200 patients with AIS admitted to the emergency room are eligible for this study. The patients will be divided into two groups: One hundred will be assigned to the experimental group and 100 to the control group. This will allow the intervention to start from day 0 of hospitalization in the neurology ward.

The inclusion and exclusion criteria are listed in Table 1.

**Table 1:** The inclusion and exclusion criteria.

**Inclusion Criteria**	**Exclusion Criteria**
Patients aged 50-80 years	Reperfusion treatment (intravenous thrombolysis, mechanical thrombectomy)
5 < NIHSS scores < 25	Hemorrhagic stroke on CT scan
Ischemic stroke confirmed by CT scan	5 > NIHSS scores > 25
<24 hours from onset to treatment	Fluctuating neurological deficit
Signed informed consent	Transient ischemic attack
	Other cerebral lesions: cerebral tumors, arteriovenous malformation.
	Uncontrolled blood pressure with systolic pressure < 90/60 mmHg or > 200/100 mmHg
	Difference between systolic blood pressure in the upper arms > 10 mmHg
	Ischemic events in the last 6 months (AIS, myocardial infarction and others)
	Premorbid Rankin >3
	Comorbidity with any serious diseases and life expectancy of less than 6 month

### Intervention

The subjects will be double-masked and randomized into the experimental and control group in a 1:1 ratio, according to the predefined drawing lots.

A manual tourniquet will be placed around the upper arm, contralateral to the neurological deficit (to reduce the possible local adverse events) in the first 24 hours after the diagnosis of AIS. All patients will receive five cycles of three-minute inflation of the blood pressure cuff, followed by five minutes of reperfusion. The procedure will be performed twice daily (morning and afternoon) during the first five days of hospitalization. In the experimental group, the level of cuff inflation will be >20 mmHg above the systolic blood pressure, up to 180 mmHg. The subjects in the control group will receive cuff inflation up to 30 mmHg, which will induce sham conditioning.

## Follow-up time

All included patients will be reevaluated clinically after three months (90 days) and 6 months (180 days) and a brain CT scan will be performed at 6 months (180 days) (Table 2).

**Table 2:** Study protocol.

**Acute Ischemic stroke** **5 <NIHSS score <25 (randomized)**	**Day 0- 5**	**90 days**	**180 days**
**RIC - experimental group**	• Guideline-based treatment • RIC twice/day • NIHSS, mRS • Infarct volume	• Guideline-based treatment • NIHSS, mRS	• Guideline-based treatment • NIHSS, mRS • Infarct volume
**RIC - control group**	• Guideline-based treatment • RIC twice/day • NIHSS, mRS • Infarct volume	• Guideline-based treatment • NIHSS, mRS	• Guideline-based treatment • NIHSS, mRS • Infarct volume

## Results

The primary outcome measure will be radiological, assessing the difference of brain infarct volume measured by CT at baseline and 180 days in the experimental group versus the control group. The CT scan will be performed at day 3-4 (or at discharge) and 180 days. The baseline infarct volume will be determined as the hypodensity image on the CT scan and measured by manual outlining. The follow-up volume at 180 days will be defined by the same method. The infarct growth will be defined as the difference between the follow-up volume and the baseline volume. The radiologist will be blinded to the clinical data, and the group examined. The same CT scan protocol will be used in both centers.

The second outcome measure will be clinical and will assess the magnitude of improvement by

1. Incidence of improvement or deterioration (not due to hemorrhage transformation) of NIHSS at 90 and 180 days compared with baseline;
2. Modified Rankin Scale (mRS), Instrumental Activities of Daily Living (IADL), Activities of Daily Living (ADL), Montreal Cognitive Assessment (MoCA), Patient Health Questionnaire-9 (PHQ-9) scores at 90 days and 180 days compared to baseline;
3. Tolerance and side effects of RIC;
4. Recurrence of stroke or other vascular events at 180 days;
5. Incidence of stroke-associated comorbidities;
6. The proportion of death by any cause during 180 days.

### Statistical analysis

The statistical analysis of the data will be conducted using SPSS 20.0 Software. Descriptive epidemiology, a correlation between risk factors and outcomes, a comparison between the two groups in terms of clinical and radiological outcomes as well as adverse effects are targeted.

Current status: at the time of the first submission of the article, 40 patients have been recruited.

## Discussion

To our knowledge, this is the first clinical trial to investigate the effect of RIC after a stroke by measuring the ischemic volume on the CT scan.

The primary outcome measure in our study will be radiological, showing the difference in brain infarct size volume between the experimental group and the control group. In the STAIR Group 2016 (Stroke Treatment Academic Industry Roundtable [4]), the infarct volume has been chosen as the radiological objective outcome, being linked to the clinical outcome [5].

The clinical outcome measure will be investigated as a second target using multiple validated scales: NIHSS, Rankin, Barthel, IADL, ADL. All these reflect the quality of life of a stroke survivor. Also, impaired scores are associated with poorer medical health, increasing medical costs. We will also investigate the cognitive status (MoCA test) and depression (PHQ-9 test) comparing the two groups at baseline, at 90 days, and 180 days.

Our study differs in some respects compared to previous ones. Firstly, it is a multicentre study, including patients from 2 hospitals. Secondly, all the patients will have an ischemic stroke confirmed on the CT scan. Previous studies assessed the brain infarct volume on MRI (REPOST study [6], RESCUE study [7], RECAST study [8], Hougaard’s study [9], Meng’s study [10], Mi’s study [11], Wang’s study [12]). Thirdly, we will include moderate and severe stroke patients (NIHSS= 5-25). Previous studies included either mild-moderate (NIHSS= 0-15) [10,13] or any stroke severity [9]. Fourthly, we will include all ischemic stroke subtypes, according to the TOAST classification. Fifthly, we will measure the final infarct volume at 180 days (6 months). Previous studies measured the final infarct volume at 24 hours [7, 9] or day 4 [6]. Finally, there are different types of RIC procedures in the previous studies: sites of limb conditioning: arm, leg, both arms, both legs; cycles and duration of inflation/deflation; timing of RIC; frequency: once or repeated /day.

## Conclusion

Remote ischemic conditioning represents a promising therapy, inexpensive, and well-tolerated, which might be neuroprotective. Provided that remote ischemic conditioning shows good results in terms of efficiency, it could be easily implanted as a routine procedure in prehospital, emergency room, all hospitals, or even intensive care units.

## Conflict of Interest

The authors declare that there is no conflict of interest.
